# G2019S selective LRRK2 kinase inhibitor abrogates mitochondrial DNA damage

**DOI:** 10.1038/s41531-024-00660-y

**Published:** 2024-03-01

**Authors:** Nicholas Pena, Tara Richbourg, Claudia P. Gonzalez-Hunt, Rui Qi, Paul Wren, Carrolee Barlow, Natalie F. Shanks, Holly J. Carlisle, Laurie H. Sanders

**Affiliations:** 1 Departments of Neurology and Pathology, Duke University School of Medicine, Durham, NC 27710 USA; 2 Duke Center for Neurodegeneration and Neurotherapeutics, Duke University, Durham, NC USA; 3 ESCAPE Bio, Inc., South San Francisco, CA 94080 USA

**Keywords:** Neurodegeneration, Parkinson's disease

## Abstract

Pathogenic mutations in *LRRK2* cause Parkinson’s disease (PD). The G2019S variant is the most common, which results in abnormally high kinase activity. Compounds that target LRRK2 kinase activity are currently being developed and tested in clinical trials. We recently found that G2019S LRRK2 causes mitochondrial DNA (mtDNA) damage and treatment with multiple classes of LRRK2 kinase inhibitors at concentrations associated with dephosphorylation of LRRK2 reversed mtDNA damage to healthy control levels. Because maintaining the normal function of LRRK2 in heterozygous G2019S *LRRK2* carriers while specifically targeting the G2019S LRRK2 activity could have an advantageous safety profile, we explored the efficacy of a G2019S mutant selective LRRK2 inhibitor to reverse mtDNA damage in G2019S LRRK2 models and patient cells relative to non-selective LRRK2 inhibitors. Potency of LRRK2 kinase inhibition by EB-42168, a G2019S mutant LRRK2 kinase inhibitor, and MLi-2, a non-selective inhibitor, was determined by measuring phosphorylation of LRRK2 at Ser935 and/or Ser1292 using quantitative western immunoblot analysis. The Mito DNA_DX_ assay, which allows for the accurate real-time quantification of mtDNA damage in a 96-well platform, was performed in parallel. We confirmed that EB-42168 selectively inhibits LRRK2 phosphorylation on G2019S LRRK2 relative to wild-type LRRK2. On the other hand, MLi-2 was equipotent for wild-type and G2019S LRRK2. Acute treatment with EB-42168 inhibited LRRK2 phosphorylation and also restored mtDNA damage to healthy control levels. We further investigated the relationship between LRRK2 kinase activity, mtDNA damage and mitophagy. Levels of mtDNA damage caused by G2019S LRRK2 were fully re-established within 2 h of a LRRK2 inhibitor wash out and recovery experiment, indicating the mtDNA damage phenotype is highly dynamic. G2019S LRRK2 mitophagy defects were not alleviated with LRRK2 kinase inhibition, suggesting that mitophagy is not mechanistically regulating LRRK2 kinase-mediated reversal of mtDNA damage in this acute timeframe. Abrogation of mtDNA damage with the mutant selective tool inhibitor EB-42168 demonstrates the potential of a precision medicine approach for LRRK2 G2019S PD. Levels of mtDNA damage may serve as a potential pharmacodynamic biomarker of altered kinase activity that could be useful for small molecule development and clinical trials.

## Introduction

Parkinson’s disease (PD) is the most common age-related movement neurodegenerative disorder. This chronic disease is characterized by progressive motor disability, non-motor symptoms and decreased quality of life. Therapeutic strategies currently available rely on dopamine replacement, but do not slow or stop the progression of the disease and can lead to motor complications. While these drugs may be useful in managing symptoms, there are no disease-modifying therapies that target the underlying pathogenic mechanisms of disease; thereby this remains a significant and urgent unmet medical need for PD patients. In 2004, coding variants were first identified in *Leucine-rich repeat kinase 2* (LRRK2), with missense mutations in *LRRK2* now established as the most common genetic cause of autosomal-dominant PD^1–5^. LRRK2 is a large and complex protein, with two functional enzymatic domains—a Ras-like GTPase and a serine-threonine kinase domain. The most frequent pathogenic *LRRK2* mutation is the Gly2019Ser (G2019S) *LRRK2* variant, which results in a modest increase in kinase activity^6–8^. Interestingly, in addition to the *LRRK2* G2019S variant, all pathogenic missense *LRRK2* mutations also augment kinase activity, displaying higher levels of the autophosphorylation Ser1292 residue of LRRK2^9–12^. Therefore, the toxic gain-of-function of LRRK2 kinase activity is strongly implicated as the cause of pathogenicity^13^. Consistent with these findings, a neuroprotective effect of LRRK2 kinase inhibitors has been demonstrated in PD-relevant cell and rodent models^14^. Thus, LRRK2 represents a promising therapeutic target for disease modification and several LRRK2 kinase inhibitors are in clinical development and/or trials.

Recently, the results of testing DNL201, a CNS-penetrant, selective, ATP-competitive, small-molecule LRRK2 kinase inhibitor in early phase human clinical trials were reported^15^. Importantly, DNL201, in humans was able to cross the blood brain barrier, based on measurements of inhibitor concentrations in cerebrospinal fluid. Concomitant blood-based markers demonstrated a dose-dependent inhibition of LRRK2 kinase by DNL201^15^. However, on-target safety liabilities for LRRK2 inhibitors have been identified in preclinical models. Several studies have shown lung and/or kidney dyshomeostasis in rodent and nonhuman primate models either lacking LRRK2 or following treatment with LRRK2 kinase inhibitors, some of which may be reversible^16–26^. Of note, lung function did not seem to be impacted at the DNL201 doses tested in single-ascending dose or multiple-ascending dose (10 days) cohorts in healthy volunteers or in patients with PD. Longer-term monitoring will be critical to assess safety of chronic dosing with LRRK2 kinase inhibitors on lung and kidney function, as well as in PD patients with clinically significant history of pulmonary and kidney disease, which are often co-morbidities in this population. Heterozygous loss-of-function variants at the *LRRK2* locus do not increase PD risk and have no apparent overt deleterious health consequences^27,28^. However, somatic *LRRK2* mutations in breast cancer are associated with high-risk features and reduced patient survival^29^, consistent with a potential risk for lung adenocarcinoma with reduced LRRK2 levels^30^, emphasizing the concern of targeting LRRK2 in humans. Additionally, since the majority of PD patients carrying the G2019S LRRK2 variant are heterozygous, a precision medicine approach of selectively reducing G2019S LRRK2 kinase activity while sparing wild-type LRRK2 physiological function could offer a potential safety advantage in this patient population.

Measuring endogenous LRRK2 kinase activity by monitoring LRRK2 autophosphorylation at serine 1292 (pSer1292) has been particularly challenging in endogenous expression systems, but can be robustly detected in overexpressed cellular models or with a fractionation-based enrichment technique in G2019S LRRK2 but not wild-type tissue^11,31,32^. The phosphorylation of downstream substrates, including Rab GTPase substrates, are difficult to measure reliably due to low stoichiometry^4,5,33,34^. The disease relevance of phospho-substrates of LRRK2, such as Rab10, is unknown, and paradoxically, Rab10 phosphorylation is not elevated with the PD G2019S LRRK2 variant, a finding reported by multiple groups^35–39^. Thus, limited markers for evaluating basal G2019S LRRK2-related kinase activity exist. The indirect but kinase conformation dependent phosphorylation site at serine 935 (pSer935) is most widely used for measuring LRRK2 kinase inhibition, despite not faithfully reflecting LRRK2 protein kinase activity^12,40,41^. Further optimizing and developing tools to measure endogenous LRRK2 kinase activity and inhibition in vivo, is a critical unmet need for future clinical trials. Mitochondrial function is significantly impacted in PD^42^. Importantly, mitochondrial DNA (mtDNA) homeostasis is disrupted in both idiopathic and familial PD cases, including those with *LRRK2* mutations, and are associated with mtDNA damage^43–50^. We found that increased mtDNA damage in PD patient-derived immune cells heterozygous for the G2019S LRRK2 mutation was abrogated following treatment with multiple classes of LRRK2 kinase inhibitors and correlated with measures of pSer935 LRRK2 dephosphorylation^44,49^. Therefore, measurement of mtDNA damage may serve as a surrogate for LRRK2 kinase activity and consequently of kinase inhibitor activity. Recently LRRK2 kinase inhibitors with significant selectivity for mutant (G2019S) LRRK2 compared to wild-type LRRK2 have been discovered, but it is unknown if these compounds have similar effects on G2019S LRRK2 dependent mtDNA damage^20,51–56^. It is not known whether the accumulation of LRRK2 G2019S-dependent mtDNA damage is solely due to the pathogenic mutant allele, or the differential stoichiometry between WT-mutant and mutant-mutant protein pairing in the LRRK2 dimer, which may impact structure and enzymatic activation. Thus, we have now extended these studies and investigated whether using a selective LRRK2 kinase inhibitor was able to reverse mtDNA damage in heterozygous G2019S cells at inhibitor concentrations shown to preferentially inhibit G2019S kinase while sparing wild-type kinase activity.

The aim of the present study was to compare the highly selective G2019S LRRK2 inhibitor (EB-42168) with the non-selective LRRK2 inhibitor (MLi-2) on mtDNA damage levels in LRRK2 models and patient cells. LRRK2 phosphorylation was examined in parallel to establish target engagement and compare these biomarker outcomes in response to the selective G2019S LRRK2 inhibitor, EB-42168. We further investigated the relationship between LRRK2 kinase activity, mtDNA damage and mitophagy. Measuring mtDNA damage together with LRRK2 phosphorylation is an innovative biomarker approach to determine LRRK2 kinase activity and may be helpful in considering the drug efficacy of compounds targeting hyperactive kinase activity in G2019S LRRK2 PD patients in the context of a clinical trial.

## Results

### PD-linked G2019S *LRRK2* variant leads to increased kinase activity and mtDNA damage

**Fig. 1:**
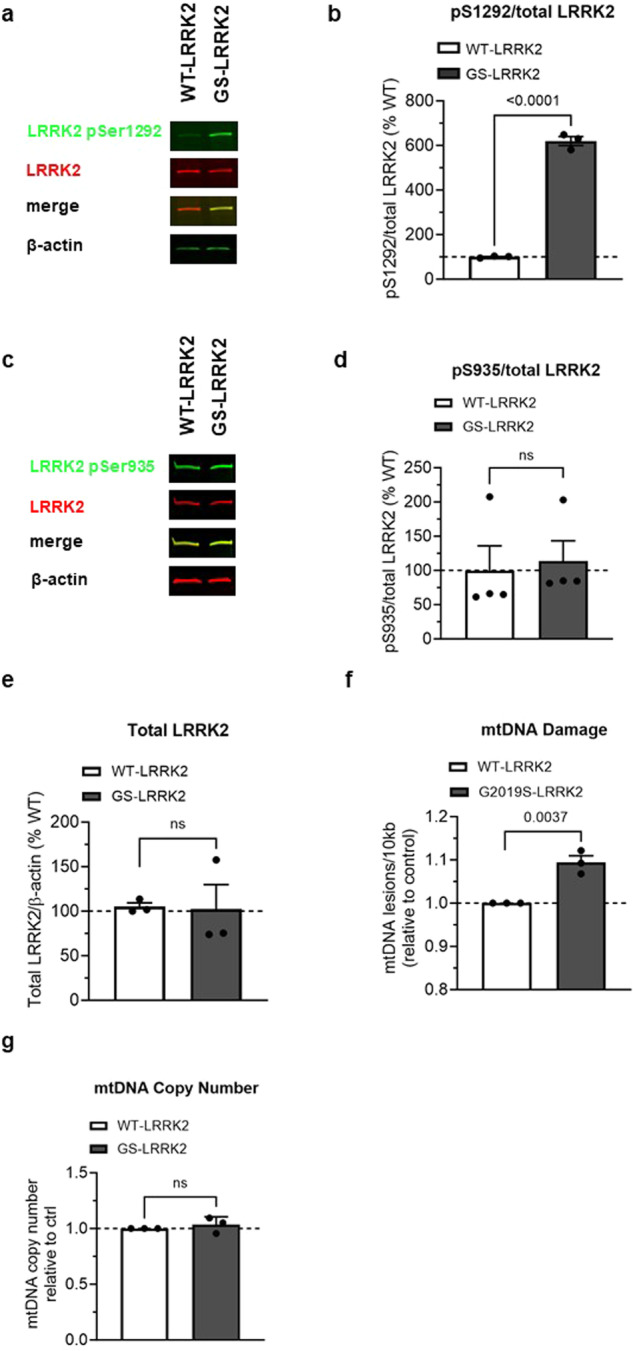
Analysis of LRRK2 and mtDNA damage in HEK293 cells lines stably overexpressing either WT-LRRK2 or G2019S-LRRK2. **a** Representative western blot of WT-LRRK2 or G2019S-LRRK2 overexpressing cells show expression of LRRK2 pSer1292 and full-length LRRK2. β-actin was blotted as a loading control. **b** Quantification of western blots demonstrate ~6-fold increase of LRRK2 pSer1292 in G2019S-LRRK2 compared to WT-LRRK2 expressing cells. **c** Representative western blots of WT-LRRK2 or G2019S-LRRK2 overexpressing cells show expression of LRRK2 pSer935 and full-length LRRK2. β-actin was blotted as a loading control. **d** Quantification of western blots demonstrate no difference of LRRK2 pSer935 levels between G2019S-LRRK2 and WT-LRRK2 expressing cells. **e** Quantification of western blots demonstrate no difference of LRRK2 protein levels between the two cell lines. **f** Mitochondrial DNA damage was increased in G2019S-LRRK2 relative to WT-LRRK2 expressing cells. **g** The differences in mtDNA damage between the cell lines were not attributable to changes in steady state mtDNA levels. Data are presented as mean ± SEM. *n* = 3–4 replicates. Statistical significance was determined by unpaired *t*-test for all analyses. GS-LRRK2 G2019S-LRRK2, WT-LRRK2 wild-type LRRK2, ns non-significant.

LRRK2 kinase activity was assessed by measuring the relative levels of autophosphorylation at pSer1292 by immunoblot in HEK293 cells stably transfected with human LRRK2 or the G2019S variant of human LRRK2 (named WT-LRRK2 and G2019S-LRRK2, respectively)^10^. pSer1292 was increased ~6 fold in G2019S-LRRK2 cells compared to WT-LRRK2, consistent with previous reports (Fig. 1a, b^20^). Levels of both pSer935 and total LRRK2 were similar between the WT-LRRK2 and G2019S-LRRK2 expressing cell lines (Fig. 1c–e). The similar expression levels of LRRK2 between the two cell lines allowed for a direct comparison of the two genotypes on mtDNA damage phenotypes. G2019S-LRRK2 induced a higher level of mtDNA damage relative to WT-LRRK2 expressing cells (Fig. 1f), without any changes in the steady state of mtDNA copy number (Fig. 1g). These results are consistent with our prior study showing that mtDNA damage was increased in primary midbrain neurons overexpressing G2019S LRRK2 compared to wild-type LRRK2, a kinase dead LRRK2 mutant or the GFP expressing control^44^.

### EB-42168 demonstrates selective inhibition of LRRK2 phosphorylation in G2019S-LRRK2 relative to WT-LRRK2 expressing cells

**Fig. 2:**
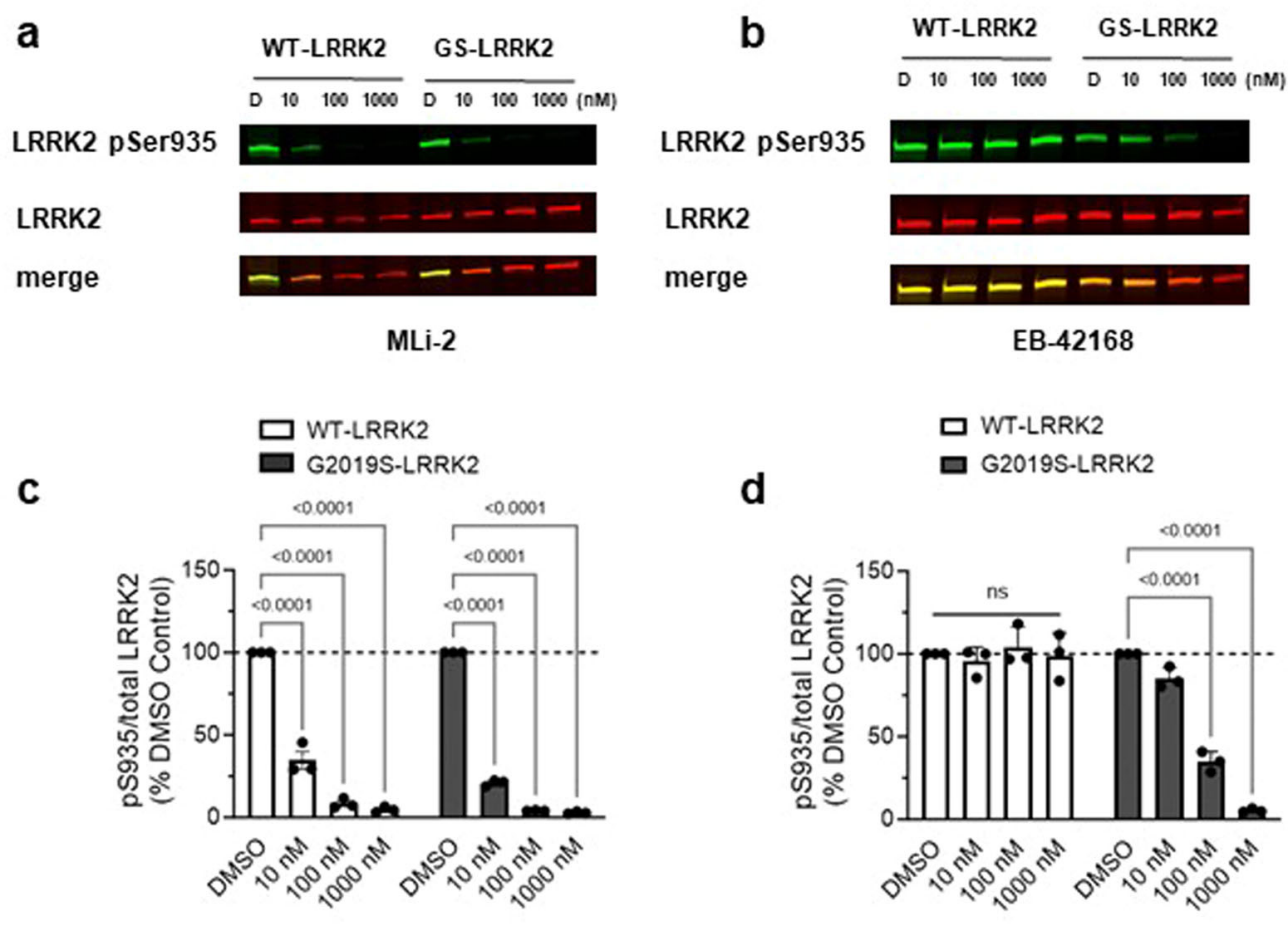
EB-42168 demonstrates full inhibition of LRRK2 pSer935 in cells overexpressing G2019S-LRRK2 at concentrations that show no inhibition in WT-LRRK2 overexpressing cells. **a** Representative western blot of WT-LRRK2 or G2019S-LRRK2 cells treated with DMSO, 10 nM, 100 nM or 1 µM MLi-2 for 2 h and assessed for LRRK2 pSer935 and full-length LRRK2. **b** Representative western blot of WT-LRRK2 or G2019S-LRRK2 cells treated with DMSO, 10 nM, 100 nM or 1 µM EB-42168 for 2 h and assessed for LRRK2 pSer935 and full-length LRRK2. **c** Quantification of western blots demonstrate a 70%, 90% and 95% decrease, respectively, in LRRK2 pSer935 levels with increasing concentration of MLi-2 in WT-LRRK2 expressing cells. Quantification of western blots demonstrate an 80%, 96% and 97% decrease respectively in LRRK2 pSer935 levels with increasing concentration of MLi-2 in G2019S-LRRK2 expressing cells. **d** Quantification of western blots demonstrate no change in LRRK2 pSer935 levels with EB-42168 treatment in WT-LRRK2 expressing cells. Quantification of western blots demonstrate a 65% and 95% decrease respectively in LRRK2 pSer935 levels with 100 nM and 1 µM of EB-42168 in G2019S-LRRK2 expressing cells. Data are presented as mean ± SEM. *n* = 3 replicates. (*p < 0.0001 determined by two-way ANOVA). GS-LRRK2 G2019S-LRRK2, WT-LRRK2 wild-type LRRK2, ns non-significant.

The LRRK2 kinase inhibitor EB-42168 was previously shown to inhibit G2019S LRRK2 100-fold more potently than wild-type LRRK2^20^. MLi‐2, by contrast, was equipotent in recombinant cell lines expressing WT-LRRK2 or G2019S-LRRK2, and can therefore serve as a non-selective LRRK2 kinase inhibitor control^20,24^. We assessed the potency of EB-42168 and MLi-2 to inhibit pSer935 and pSer1292 LRRK2 in WT or G2019S- LRRK2 expressing cells in compound titration experiments with concentrations ranging from 10 nM–1 μM. At the lowest concentration of MLi-2 tested (10 nM), pSer935 was significantly decreased by ~75% in both the WT-LRRK2 and G2019S-LRRK2 cells (Fig. 2a, b). At concentrations higher than 100 nM, MLi-2 showed nearly complete ablation of phosphorylation at Ser935, with <10% signal remaining (Fig. 2a, b). Overall, the concentration-response profile for MLi-2 at pSer935 was similar for the two genotypes, with calculated half-maximal inhibition (IC_50_) values of 4.6 and 2.3 nM in WT and G2019S LRRK2 cell lines, respectively (Fig. 2a, b). In comparison, EB-42168 inhibited pSer935 in G2019S-LRRK2 expressing cells by over 50% at 100 nM with full inhibition at 1 μM (Fig. 2c, d) but showed no inhibition of pSer935 in WT-LRRK2 cells up to 1 μM (Fig. 2c, d). The calculated IC_50_ values for EB-42168 on pS935 was >5000 nM in WT LRRK2 cells and 54 nM in G2019S LRRK2 cells. Therefore, EB-42168 was >90-fold more potent on G2019S LRRK2 which is in line with previous reports^20,51^.

**Fig. 3:**
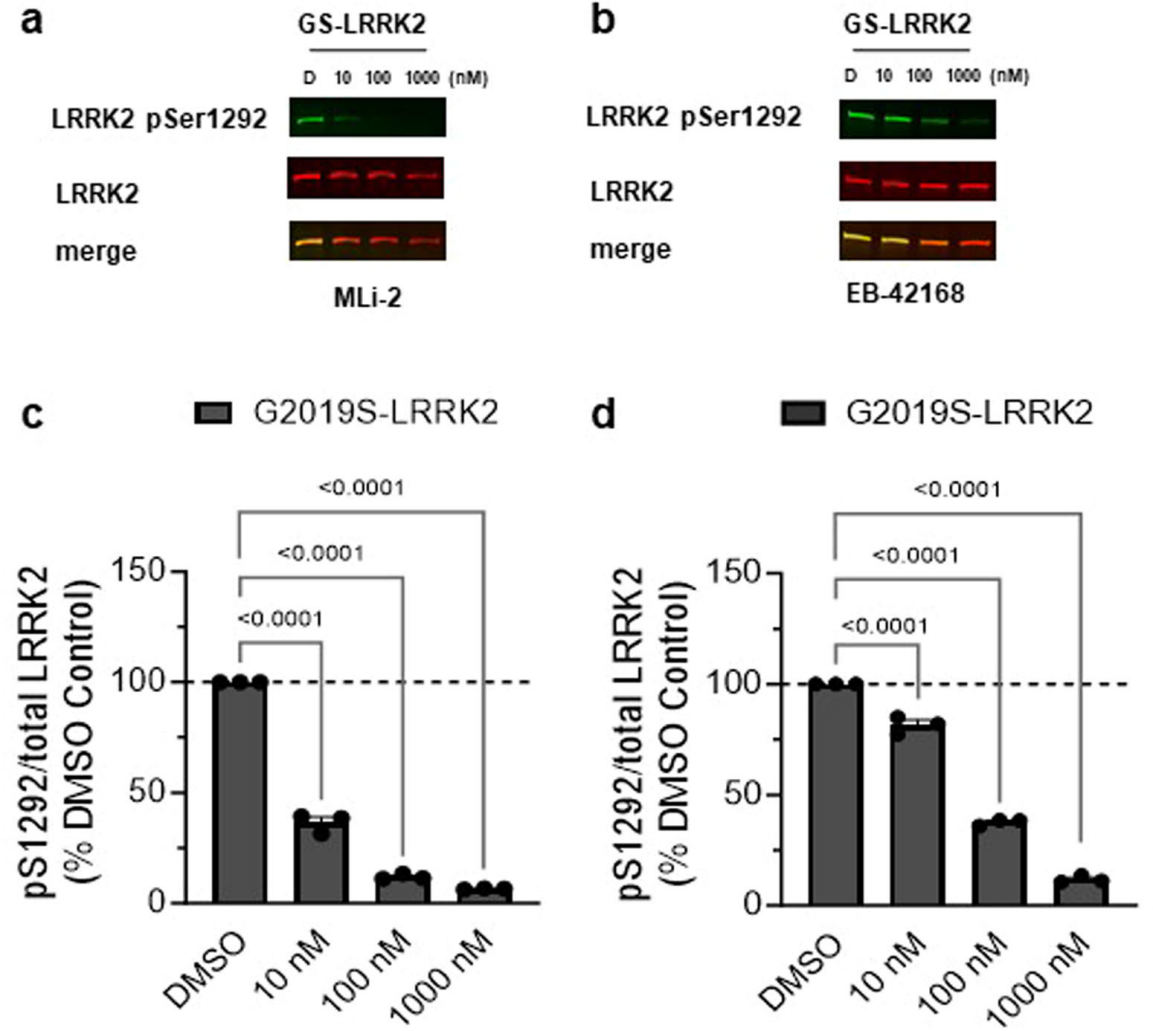
EB-42168 inhibits LRRK2 pSer1292 in G2019S-LRRK2 overexpressing cells at concentrations that also inhibited pSer935. **a** Representative western blot of G2019S-LRRK2 cells treated with DMSO, 10 nM, 100 nM or 1 µM MLi-2 for 2 h and assessed for LRRK2 pSer1292 and full-length LRRK2. **b** Representative western blot of G2019S-LRRK2 cells treated with DMSO, 10 nM, 100 nM or 1 µM EB-42168 for 2 h and assessed for LRRK2 pSer1292 and full-length LRRK2. **c** Quantification of western blots demonstrate a 65%, 90% and 95% decrease in LRRK2 pSer1292 levels with increasing concentration of MLi-2 in G2019S-LRRK2 expressing cells. **d** Quantification of western blots demonstrate a 30%, 65% and 85% decrease in LRRK2 pSer1292 levels with 10 nM, 100 nM and 1 µM respectively of EB-42168 in G2019S-LRRK2 expressing cells. Data are presented as mean ± SEM. *n* = 3 replicates. (*p < 0.0001 determined by one-way ANOVA). GS-LRRK2 G2019S-LRRK2.

MLi-2 inhibited autophosphorylation at pSer1292 at a similar potency as pSer935 in G2019S-LRRK2 expressing cells. pSer1292 levels were significantly decreased following 10 nM treatment with MLi-2 (Fig. 3a, b) and minimal pSer1292 levels remained at concentrations 100 nM and higher, with a calculated IC_50_ value of 4.7 nM (Fig. 3a, b). EB-42168 also showed similar potency on pSer1292 compared to pSer935 inhibition at higher concentrations, with ~70% reduction observed at 100 nM and full inhibition at 1 μM translating at a calculated IC_50_ value of 44 nM (Fig. 3c, d). Due to the very low basal levels of pSer1292 in WT-LRRK2 cells, inhibition of the pSer1292 could not be accurately quantified using the immunoblot method in the WT-LRRK2 cell line and therefore genotype selectivity could not be assessed.

### Acute treatment with G2019S-selective kinase inhibition mitigates G2019S LRRK2-induced mtDNA damage

**Fig. 4:**
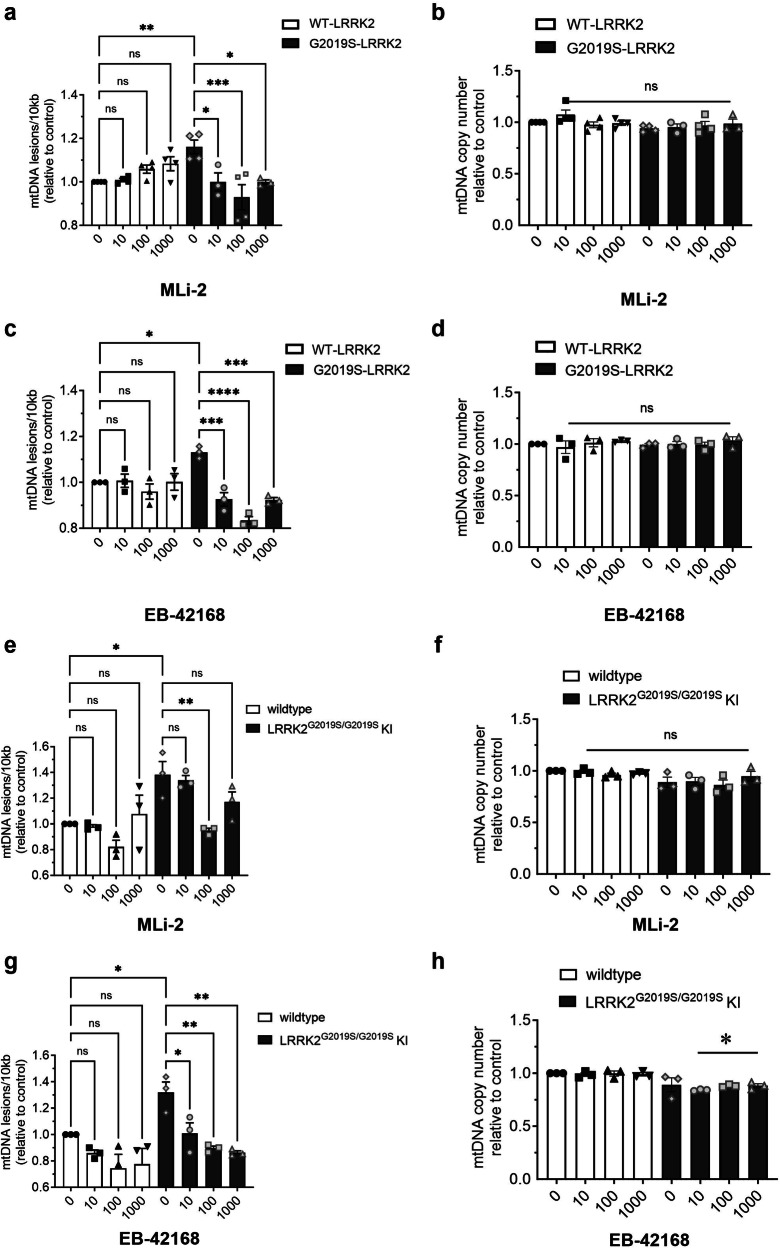
Mitochondrial DNA damage in G2019S-LRRK2 overexpression and LRRK2^G2019S/G2019S^ KI cells was abrogated to normal levels with LRRK2 kinase inhibition. **a** WT-LRRK2 and G2019S-LRRK2 cells were treated with DMSO, 10 nM, 100 nM or 1 µM MLi-2 for 2 h and analyzed for mtDNA damage. **b** mtDNA copy number was unaltered by the MLi-2 treatment. **c** WT-LRRK2 and G2019S-LRRK2 cells were treated with DMSO, 10 nM, 100 nM or 1 µM EB-42168 for 2 h and analyzed for mtDNA damage. **d** Treatment with EB-42168 did not change mtDNA copy number in either WT-LRRK2 or G2019S-LRRK2 cells. **e** LRRK2^G2019S/G2019S^ KI cells and wild-type cells were treated with DMSO, 10 nM, 100 nM or 1 µM MLi-2 for 2 h and analyzed for mtDNA damage. **f** MLi-2 treatment did not change mtDNA copy number in either cell line. **g** LRRK2^G2019S/G2019S^ KI cells and wild-type cells were treated with DMSO, 10 nM, 100 nM or 1 µM EB-42168 for 2 h and analyzed for mtDNA damage. **h** In contrast, while EB-42168 had no effect on mtDNA copy number in wild-type cells, mtDNA copy number was modestly decreased in EB-42618 treated LRRK2^G2019S/G2019S^ KI cells. Data are presented as mean ± SEM. *n* = 3 replicates. (*p < 0.05, **p < 0.01, ***p = 0.0001, ****p < 0.0001 determined by one-way ANOVA). GS-LRRK2 G2019S-LRRK2, WT-LRRK2 wild-type LRRK2, ns non-significant.

The loss of constitutive phosphorylation of LRRK2 at Ser935 in the presence of a LRRK2 kinase inhibitor is rapid (refs. 57,58, Figs. 2 and 3). We recently reported that acute treatment of G2019S LRRK2 patient-derived cells to LRRK2 kinase inhibitors restored mtDNA damage to control levels^44,49^. To determine whether the time course of mtDNA damage reversal by LRRK2 kinase inhibitors also occurs quickly with a G2019S-selective inhibitor, WT-LRRK2 or G2019S-LRRK2 expressing cells were exposed to MLi-2, EB-42168, or vehicle for 2 h. Treatment of G2019S-LRRK2 cells to MLi-2 restored mtDNA damage to WT-LRRK2 control levels at concentrations ranging from 10 nM to 1 μM (Fig. 4a). Culturing in the presence of MLi-2 at higher concentrations induced a trend toward an increase in mtDNA damage in WT-LRRK2 cells (Fig. 4a). MLi-2 had no effect on mtDNA copy number in WT-LRRK2 and G2019S-LRRK2 cells (Fig. 4b). Interestingly, all concentrations of EB-42168 tested alleviated G2019S-LRRK2 induced mtDNA damage, normalizing damage to WT-LRRK2 control levels (or lower) within 2 h of compound addition (Fig. 4c). No effect of EB-42168 was observed in WT-LRRK2 cells at the range of concentrations tested (Fig. 4c). Mitochondrial DNA copy number was not different in G2019S-LRRK2 or WT-LRRK2 cells following an acute treatment with EB-42168 (Fig. 4d).

To investigate the effect of MLi-2 and EB-42168 in cells with endogenous levels of LRRK2, we utilized homozygous G2019S knock-in (LRRK2^G2019S/G2019S^ KI) HEK293 cells generated by CRISPR/Cas9 gene editing^32^. Mitochondrial DNA damage was statistically significantly increased in LRRK2^G2019S/G2019S^ KI cells compared to wild-type cells (Fig. 4e), without a change in mtDNA copy number (Fig. 4f). In LRRK2^G2019S/G2019S^ KI cells, a 100 nM concentration of MLi-2 reversed mtDNA damage levels to wild-type levels (Fig. 4e). No effect was observed on mtDNA copy number (Fig. 4f). In contrast, mtDNA damage in LRRK2^G2019S/G2019S^ KI cells were reversed to wild-type levels at all EB-42168 concentrations tested (Fig. 4g). Interestingly, mtDNA copy number was reduced in EB-42168 treated LRRK2^G2019S/G2019S^ KI cells (Fig. 4h).

### G2019S LRRK2-induced mtDNA damage is dynamic

**Fig. 5:**
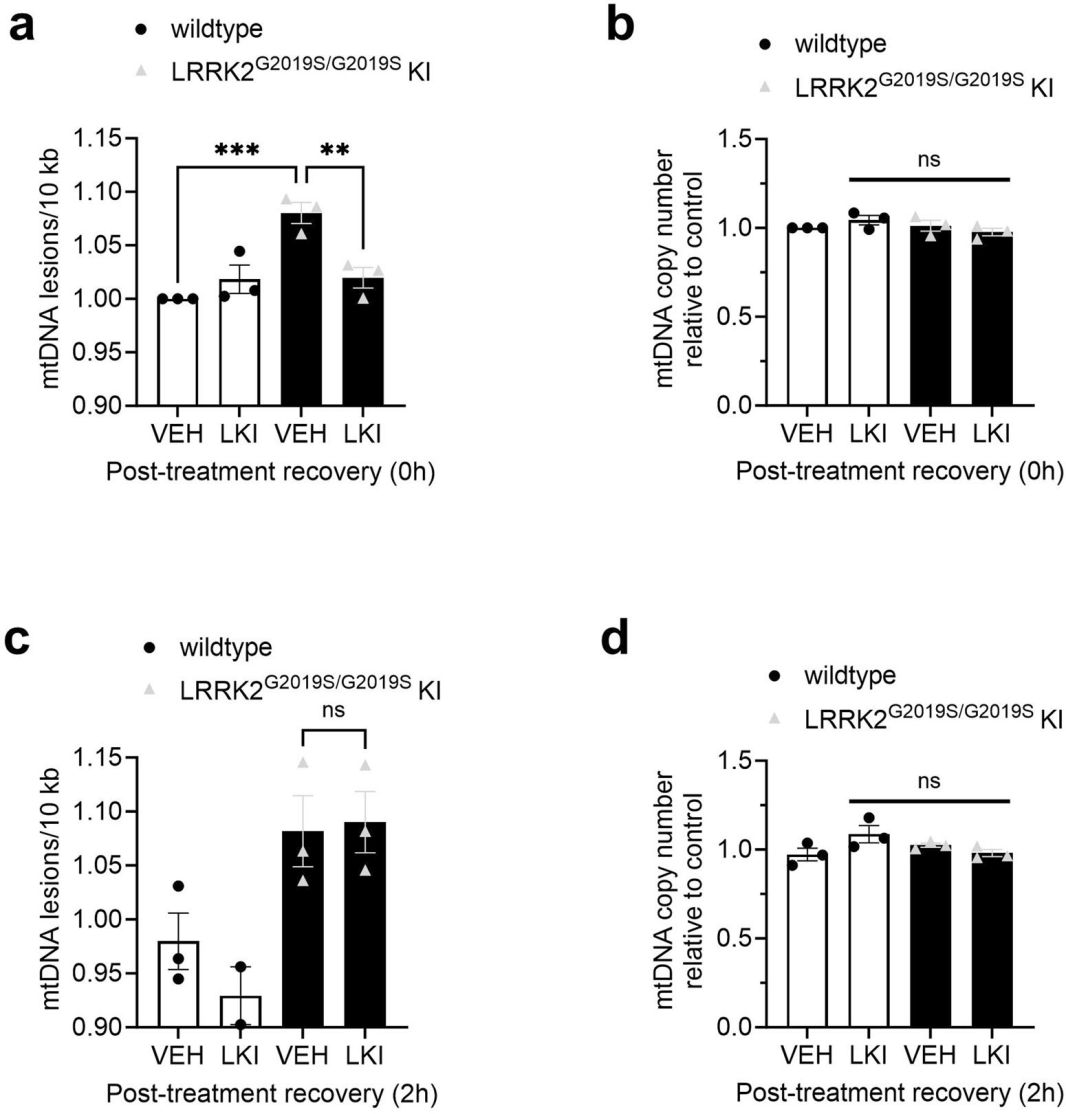
G2019S LRRK2-dependent mtDNA damage is re-established within 2 h following washout of LRRK2 kinase inhibitor. **a** Wild-type and LRRK2^G2019S/G2019S^ KI cells were treated with vehicle or 100 nM MLi-2 for 2 h and analyzed for mtDNA damage. **b** mtDNA copy number was not affected by experimental conditions. **c** Wild-type and LRRK2^G2019S/G2019S^ KI cells were treated with vehicle or 100 nM MLi-2 for 2 h, followed by washout and incubation in fresh media for 2 h and analyzed for mtDNA damage. **d** mtDNA copy number did not change with treatment condition. Data are presented as mean ± SEM. *n* = 2–3 replicates. (**p < 0.01, ***p < 0.001 determined by one-way ANOVA with a Bonferroni’s multiple comparisons test). ns non-significant.

Given the acute nature of the restoration of mitochondrial genome integrity with LRRK2 kinase inhibition, we further tested the rapid and dynamic nature of the mtDNA damage phenotype. LRRK2^G2019S/G2019S^ KI and wild-type cells were treated for 2 h with vehicle or MLi-2, and then either collected immediately or was followed by a washout into media allowing 2 h of recovery. When cells were treated with vehicle or MLi-2 and collected immediately, mtDNA damage was increased in LRRK2^G2019S/G2019S^ KI cells and 100 nM concentration of MLi-2 reversed mtDNA damage levels to wild-type levels (Fig. 5a); consistent with data presented in Fig. 4e. No changes were observed with mtDNA copy number (Fig. 5b). Interestingly, when cells were treated with vehicle or MLi-2 for 2 h and then allowed to recover in media, the mtDNA damage phenotype is fully re-established within 2 h following the wash out (Fig. 5c), with no changes noted in mtDNA copy number (Fig. 5d).

**Fig. 6:**
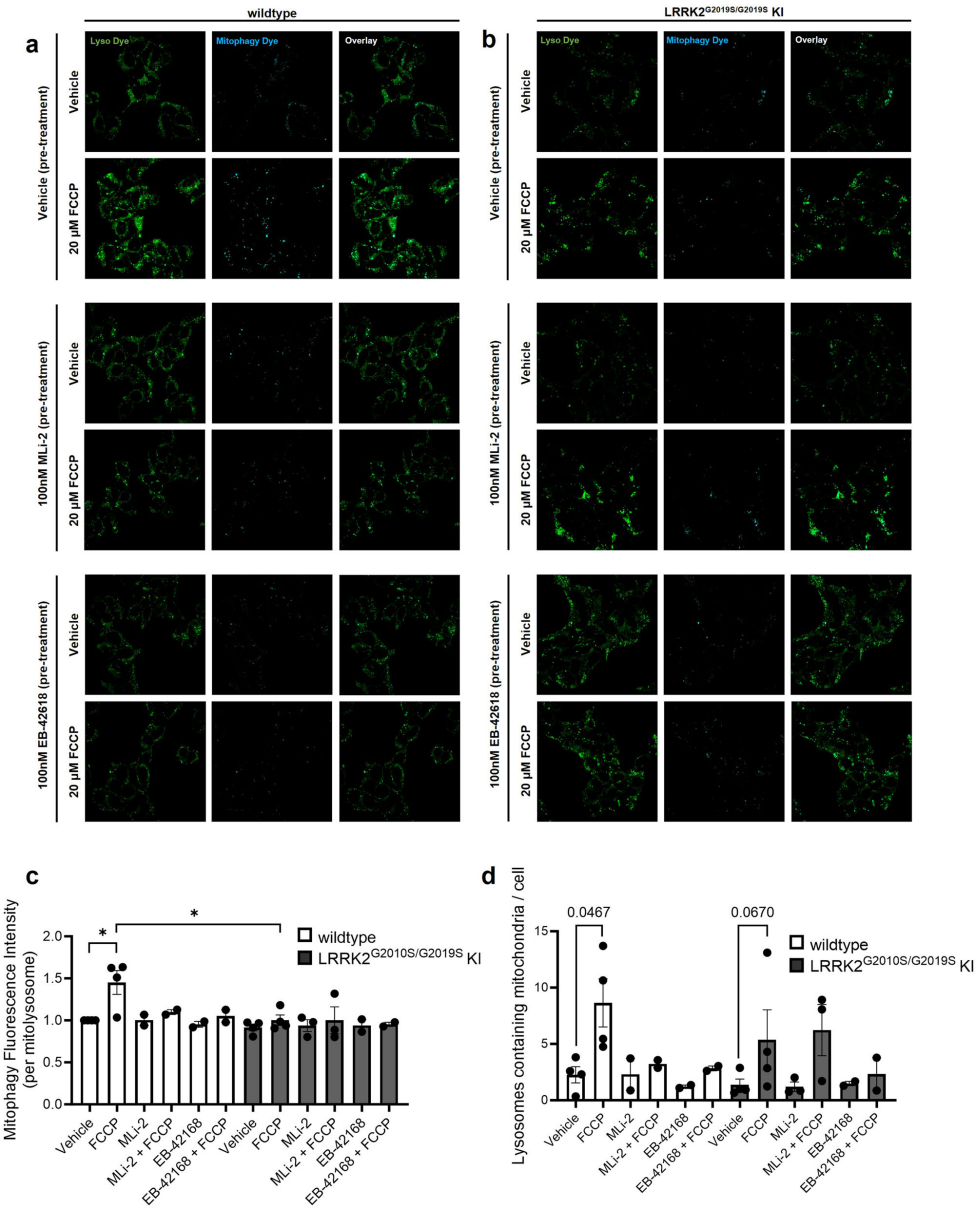
Basal and depolarization-induced mitophagy in wild-type and LRRK2^G2019S/G2019S^ KI cells pre-treated with vehicle or LRRK2 kinase inhibitor. Representative images of (**a**) wild-type or (**b**) LRRK2^G2019S/G2019S^ KI cells with the Lyso and Mitophagy dye with a 2 h pre-treatment with either 100 nM MLi-2, EB-42168, or vehicle, followed by a 3 h treatment with 20 μM FCCP or vehicle. **c** Quantification of pH-based Mitophagy dye fluorescence intensity within mitolysosomes at baseline and under FCCP-induced mitophagy. **d** Quantification of the number of lysosomes containing fluorescing mitochondria normalized by cell count. Data are presented as mean ± SEM. *n* = 2–4 replicates. (*p < 0.05 determined by one-way ANOVA).

We next investigated the temporal relationship between LRRK2 kinase activity and mitophagy. To measure mitophagy we employed a pH-sensitive Mitophagy Dye, which binds to mitochondria prior to induction of mitophagy and fluoresces at low levels at cytosolic pH (see “Methods” for a full detailed description). Therefore, the fluorescence intensity of mitochondria-bound Mitophagy Dye will increase upon introduction to an acidic environment such as a lysosome. Localization of mitochondria within lysosomes is confirmed using the Lyso Dye. LRRK2^G2019S/G2019S^ KI and wild-type cells were pre-treated for 2 h with vehicle, MLi-2 or EB-42168 and then challenged with FCCP or vehicle for 3 h and then mitophagy assessed (Fig. 6a, b). Two different methods to analyze mitophagy were used. First, the fluorescence intensity of the Mitophagy dye within mitolysosomes, allowed analysis of effective uptake of mitochondria into a functional, acidified lysosome (Fig. 6c). The second method leveraged analyzing co-localization of the Mitophagy and Lyso Dye, which indicates mitochondrial engulfment into lysosomes (Fig. 6d). With both methods of analyses, mitophagy was detectable in wild-type cells; however basal levels of mitophagy in LRRK2^G2019S/G2019S^ KI cells did not differ from wild-type (Fig. 6a–d). This was not due to changes in overt mitochondrial network alterations (Supplementary Fig. 1) or mtDNA copy number (Fig. 4). While mitophagy was increased following treatment with the potent mitophagy inducer FCCP in wild-type cells (Fig. 6a, c, d), the LRRK2^G2019S/G2019S^ KI cells displayed a defect in FCCP-stimulated mitophagy (Fig. 6b–d).

Treatment with either MLi-2 or EB-42618 did not impact basal levels of mitophagy in wild-type or LRRK2^G2019S/G2019S^ KI cells, measured by Mitophagy dye fluorescent intensity or co-localization (Fig. 6a–d). FCCP-stimulated mitophagy was blocked by both MLi-2 and EB-42618 in wild-type cells (Fig. 6c, d). Neither the non-selective or selective LRRK2 kinase inhibitor rescued the FCCP-stimulated mitophagy defect in LRRK2^G2019S/G2019S^ KI cells (Fig. 6c, d).

### EB-42168 demonstrates selective inhibition in heterozygous G2019S LRRK2 PD patient-derived cells compared to healthy controls

**Fig. 7:**
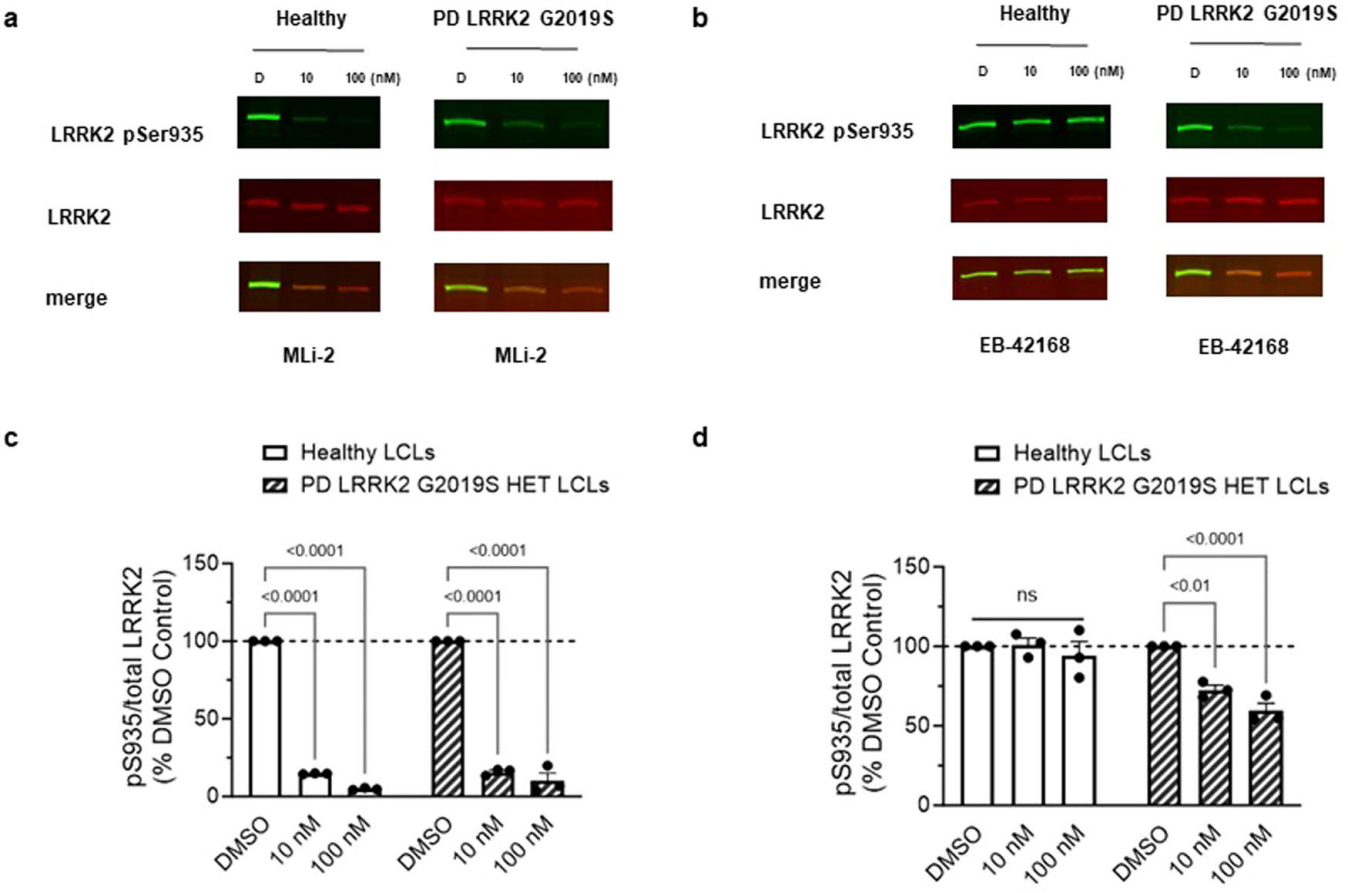
EB-42168 demonstrates selective inhibition of pSer935 LRRK2 in heterozygous G2019S LRRK2 PD patient-derived LCLs relative to healthy controls. **a** Representative western blot of PD patient-derived LRRK2 G2019S carriers and age-matched healthy controls LCLs treated with DMSO, 10 nM, or 100 nM MLi-2 for 2 h and assessed for LRRK2 pSer935 and full-length LRRK2. **b** Representative western blot of PD patient-derived LRRK2 G2019S carriers and age-matched healthy controls LCLs treated with DMSO, 10 nM, or 100 nM EB-42168 for 2 h and assessed for LRRK2 pSer935 and full-length LRRK2. **c** Quantification of western blots demonstrate an 85% and 95% decrease in LRRK2 pSer935 levels in MLi-2 treated LRRK2 G2019S PD patient-derived and age-matched healthy controls. **d** Quantification of western blots demonstrate no change in LRRK2 pSer935 levels with EB-42168 treatment in healthy controls. Quantification of western blots demonstrate a 25% and 40% decrease in LRRK2 pSer935 levels with 10 nM and 100 nM EB-42168 in PD patient-derived LRRK2 G2019S carriers. Data are presented as mean ± SEM. (**p < 0.01 determined by two-way ANOVA).

To investigate the selective effect of EB-42168 relative to MLi-2 on LRRK2 phosphorylation and mtDNA damage biomarkers in PD patient cells, LRRK2 G2019S and healthy control-derived lymphoblastoid cells (LCLs) were evaluated. Of note, only LRRK2 pSer935 levels were measured in response to LRRK2 kinase inhibition, as the pSer1292 signal in LCLs was not quantifiable by immunoblotting methods^11,49^. MLi-2 at 10 and 100 nM reduced pSer935 almost completely in both healthy controls and G2019S LRRK2 PD patient-derived LCLs, similar to previously published results (Fig. 7a, c^49^). Treatment with EB-42168 did not impact pSer935 levels in healthy controls (Fig. 7b, d). In contrast, in heterozygous G2019S LRRK2 PD patient-derived LCLs, treatment with EB-42168 partially reduced LRRK2 pSer935 phosphorylation, ~30 and 40% with 10 and 100 nM, respectively (Fig. 7b, d), presumably due to a lack of sensitivity on WT LRRK2 which accounts for ~50% of the total pSer935 signal in heterozygous cells.

**Fig. 8:**
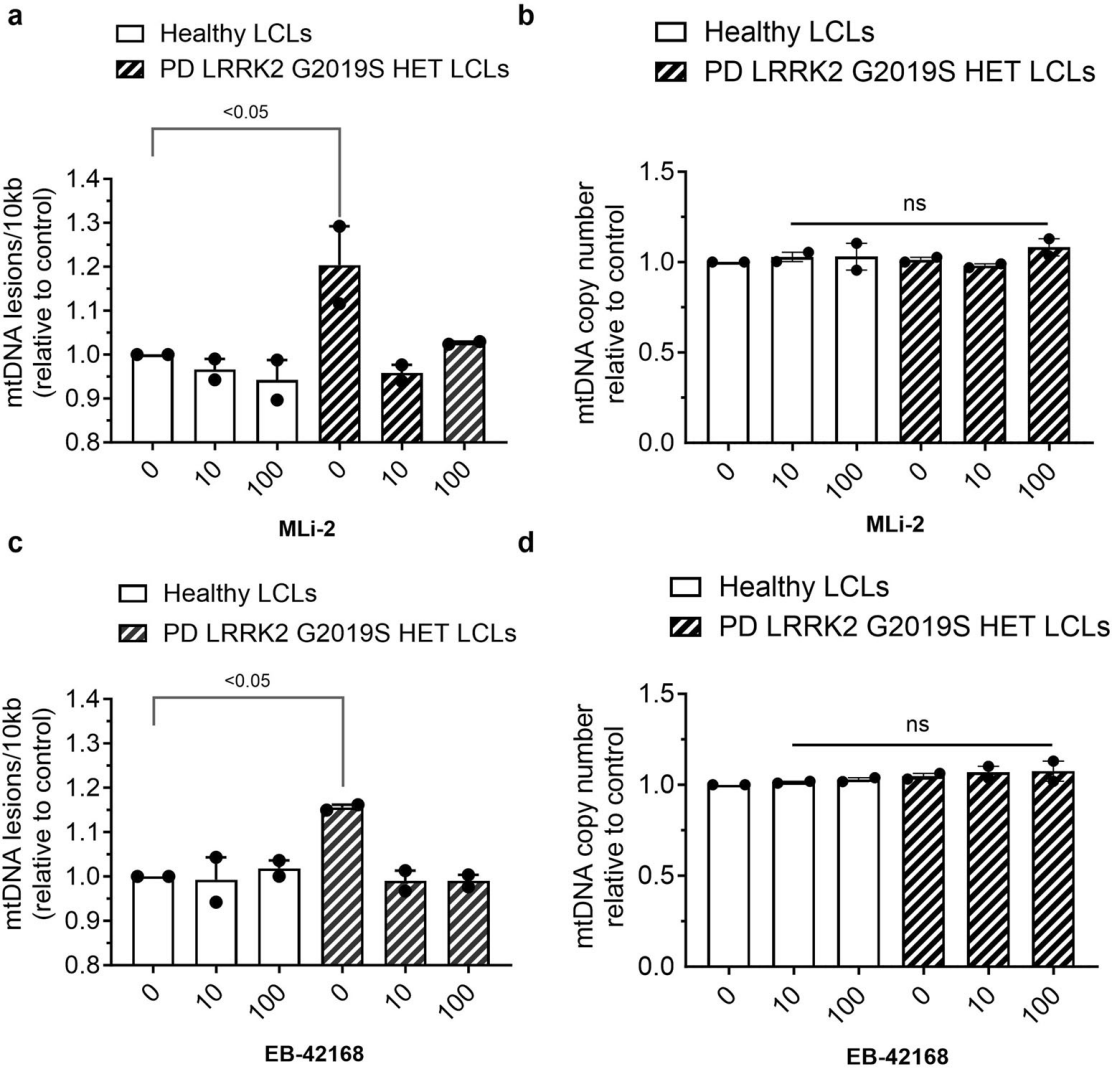
LRRK2 kinase inhibitor treatment restored LRRK2 G2019S induced mtDNA damage to basal healthy control levels. **a** PD patient-derived heterozygous G2019S LRRK2 carriers and age-matched healthy controls LCLs treated with DMSO, 10 nM, or 100 nM MLi-2 or (**c**) EB-42168 for 2 h and analyzed for mtDNA damage. **b**, **d** mtDNA copy number was unaltered by these treatments. Data are presented as mean ± SEM. (*p < 0.05 determined by one-way ANOVA).

Mitochondrial DNA damage levels were measured in parallel. Acute treatment with MLi-2 similarly reversed mtDNA damage in G2019S LRRK2 patient-derived LCLs at all concentrations tested, but had no effect in healthy controls (Fig. 8a). No differences in mtDNA copy were detected with MLi-2 treatment, regardless of PD status or genotype (Fig. 8b). Importantly, similar results were found with EB-42168: mtDNA damage was restored to control levels at concentrations of 10 and 100 nM in heterozygous G2019S LRRK2 LCLs, without an effect in healthy controls (Fig. 8c). No differences in mtDNA copy were detected with EB-42168 treatment in either healthy controls or PD patient-derived G2019S LRRK2 LCLs (Fig. 8d).

## Discussion

Significant progress has been made elucidating cellular pathways that are altered by kinase-activating LRRK2 variants associated with familial and idiopathic PD. Previously, we demonstrated that mtDNA damage is increased specifically in dopaminergic neurons in human PD patient-derived post-mortem brains and in vivo PD models^43^. Mitochondrial DNA damage is elevated in PD patient-derived LRRK2 mutant neurons and immune cells and is dependent on LRRK2 kinase activity^39,43–45,49^. We further demonstrated that mtDNA damage in human heterozygous G2019S LRRK2 PD patient-derived cells was restored to healthy control levels following treatment with LRRK2 kinase inhibitors in a dose-dependent fashion^49^. The objective of the current study was to investigate whether G2019S LRRK2 dependent mtDNA damage could also be abrogated by selectively inhibiting only pathogenic LRRK2 kinase activity derived from the G2019S LRRK2 mutant allele. Our data demonstrates that mtDNA damage is induced by the PD-associated G2019S mutation in LRRK2, either in an overexpressed context or KI model, thus indicating that the G2019S mutant expressed at endogenous levels is sufficient to drive and cause mtDNA damage in vitro. We further illustrate that the mtDNA damage phenotype can be restored by pharmacological treatments with both selective and non-selective LRRK2 inhibitors in LRRK2 cellular models and G2019S LRRK2 PD patient-derived cells. Together, these data indicate that mtDNA damage can serve as a functional pharmacodynamic marker for G2019S-selective and non-selective LRRK2 kinase inhibition and provide critical insight into LRRK2-based therapeutics.

*LRRK2* pathogenic mutations, including the G2019S variant, lead to hyperactive kinase activity^6–8^. As such, LRRK2-associated PD medicinal chemistry programs have focused on the development of LRRK2 kinase inhibitors. While these inhibitors have high specificity for the kinome, most do not discriminate between WT-LRRK2 and pathogenic G2019S-LRRK2. The strategy of pursuing specific G2019S-LRRK2 kinase inhibitors may be critical in mitigating potential safety liabilities associated with the impact of non-selective LRRK2 kinase inhibitors on the wild-type allele. In this pursuit, the first identification of small molecules that could precisely target a mutation in LRRK2 was discovered in a high-throughput screen, of which the scaffold was further optimized for a series of novel, potent compounds that preferentially inhibited G2019S-LRRK2^20^. Utilizing one of these compounds, EB-42168, we found with increasing concentrations that LRRK2 dephosphorylation was only observed in G2019S-LRRK2 expressing cells with no detectable effect on WT-LRRK2. These results are consistent with previous findings that EB-42168 inhibited G2019S-LRRK2 100-fold more potently than WT-LRRK2^20^. In human PD patient-derived cells carrying a heterozygous G2019S LRRK2 mutation, we found that LRRK2 pSer935 was decreased about 40%, without a change in healthy controls. Importantly, the effect of EB-42168 was similar in an artificial overexpression system and in human derived cells with endogenous levels of human LRRK2. Similar levels of inhibition of pSer935 LRRK2 with EB-42168 in heterozygous G2019S LRRK2 carriers derived from human peripheral blood mononuclear cells has been reported^51^. On the other hand, a non-selective inhibitor, MLi-2 at 10 nM and above, demonstrated almost complete pSer935 LRRK2 inhibition, in both G2019S and WT-LRRK2 overexpression systems and in human derived healthy controls and G2019S LRRK2 PD patient cells; not surprisingly based on previous findings^44,49,51^. In addition to EB-42168, novel structural and G2019S-selective LRRK2 inhibitors have recently been reported^52,53^. Critical next steps include exploring the effect of different structural classes of G2019S-selective LRRK2 inhibitors on levels of mtDNA damage. Although EB-42168 is a poorly brain penetrant molecule^20^, G2019S-selective LRRK2 inhibitors with promising pharmacokinetic properties have been identified^52,54,56^. Testing brain penetrant G2019S-LRRK2 selective compounds in vivo on LRRK2 phosphorylation and mtDNA damage biomarkers will be explored in future studies.

While the rationale for targeting pathogenic LRRK2 is compelling, the precise amount of kinase inhibition that is needed to be potentially efficacious remains to be elucidated. This is partially due to the inherent challenges in using surrogate markers of activity. Current potential brain and peripheral biomarkers of LRRK2 kinase activity are either indirect, lack sensitivity or do not correlate with intrinsic cellular kinase activity of LRRK2, posing challenges to the development of LRRK2-targeted therapeutics^35,59–63^. It is also unclear that the current biomarkers can capture the dynamic nature of LRRK2 kinase activity and whether brain and peripheral changes are equivalent comparisons. Biomarkers such as mtDNA damage are found both in peripheral (replicating) and neurons (post-mitotic cells), suggesting this phenotype is robust across some cell-types and may reflect neuronal processes. Though it remains to be elucidated whether the underlying mechanism(s) causing the accumulation of mtDNA damage are similar in neurons vs. peripheral tissues, which is critical to understand mechanisms related to mitochondrial dysfunction in the brain and how (or not) this relates to the biology in the periphery. Interestingly, here we found that <50% inhibition with both MLi-2 and EB-42618 was required to abrogate mtDNA damage in an endogenous expression system. These findings are consistent with our previous report in which we found with non-selective LRRK2 kinase inhibitors that ~25% inhibition of LRRK2 pSer935 was sufficient to reverse mtDNA damage levels back to healthy control levels^49^. However, we were not able to reliably measure pSer1292 LRRK2 under the same conditions, a barrier in most endogenous contexts^31^. An advantage to the current study is that we utilized stably overexpressing LRRK2 cell lines to compare how pSer1292 and pSer935 LRRK2 dephosphorylation changes with non-selective and selective LRRK2 kinase inhibitors. At the MLi-2 concentrations tested, LRRK2 pSer935 and pSer1292 had similar trends and equal levels of dephosphorylation. However, to our surprise, at 10 nM of EB-42618, no significant dephosphorylation of LRRK2 pSer935 was detected, yet LRRK2 pSer1292 was dephosphorylated, indicating that LRRK2 kinase activity was inhibited. Even without target engagement of pSer935 LRRK2 at 10 nM with EB-42618 by immunoblotting, mtDNA damage was abrogated, highlighting that mitochondrial genome integrity is extremely sensitive and may accurately reflect intrinsic LRRK2 kinase activity^12,40,41^. The majority of preclinical studies have evaluated neuroprotection or related PD-pathology with >90% pSer935 inhibition^64^. Yet, only partial inhibition of LRRK2 kinase activity could be sustained without lung pathology in monkeys^22^. Therefore, future preclinical and clinical studies may consider evaluating these multiple biomarkers, and levels of mtDNA damage in human blood hold promise for guiding this balancing act of efficacy and minimizing safety risk^39^.

While mitochondrial dysfunction is a prominent etiologic factor in PD^65^, the mechanism(s) driving mtDNA damage or the LRRK2 kinase mediated reversal has remained elusive^39,44,45,49^. In this study we have gained critical insights into the underlying mechanism of how LRRK2 kinase inhibitors restore G2019S LRRK2-induced mitochondrial genome homeostasis. First, the dose-response for the suppression of LRRK2 kinase mediated mtDNA damage in some cases was “U” shaped. Our data suggests that too much LRRK2 kinase inhibition can drive mtDNA damage levels, consistent with findings that maximal LRRK2 kinase inhibition has been shown to be deleterious^19^. Therefore, levels of mtDNA damage are a compelling pharmacodynamic biomarker, measuring the cellular response to LRRK2 kinase inhibitors. The mtDNA damage response is distinct from the LRRK2 pSer935 and pSer1292 phosphorylation biomarkers, that instead demonstrate a sigmoidal dose-response relationship and serve as robust target engagement markers of LRRK2 kinase inhibitors^40,66^. Our recent studies demonstrated that while the hyperactive LRRK2 G2019S kinase mutant caused an increase in mtDNA damage, the opposite effect with the LRRK2 knockout was observed in which decreased levels of mtDNA damage occurred when compared to the transgenic controls^39^. Overall, our findings emphasize that the balance of LRRK2 kinase function is critical for maintaining mitochondrial genome homeostasis and supports a complex rheostat mechanism underlying the relationship of mtDNA damage and LRRK2 kinase activity.

Our second observation that provides insight into the underlying mechanism of LRRK2 kinase mediated reversal of G2019S LRRK2 dependent mtDNA damage, is how quickly this restoration occurred and is re-established. We previously demonstrated that acute treatment (1.5 h) with the non-selective LRRK2 kinase inhibitor, MLi-2, in PD patient G2019S LCLs restored mtDNA damage to healthy control levels^49^. In the current work, we found those results of acute reversal of mtDNA damage following LRRK2 kinase inhibition to be reproducible in PD patient-derived G2019S LCLs and extended to other models of LRRK2 PD and the selective LRRK2 kinase inhibitor, EB-42618. Consistent with how quickly the reversal of G2019S LRRK2-induced mtDNA damage occurred, the wash out and recovery experiments revealed similar kinetics (i.e., within 2 h) of the re-establishment of the mtDNA damage phenotype. These results highlight the dynamic nature of LRRK2-dependent mtDNA damage.

There are multiple cellular pathways to maintain mitochondrial homeostasis, including mitophagy—the removal of damaged mitochondria through autophagy. Mitophagy is a vital process for the maintenance of mitochondrial integrity; it is a key source of quality control which ultimately serves to prevent aberrant reactive oxygen species (ROS) accumulation, detrimental compromise of ATP production, and accumulation of mtDNA lesions^67^. Thus, in considering potential pathways responsible for the rescue of mtDNA damage with LRRK2 kinase inhibitors, we explored mitophagic clearance of defective, mtDNA lesion-containing mitochondria as a candidate for this mechanism^68^. We found using a chemical induction of mitochondrial depolarization with FCCP (a well-established and potent mitophagy stimulus), a mitophagy defect in LRRK2^G2019S/G2019S^ cells; however basal levels of mitophagy were similar to wild-type cells. There are conflicting reports as to whether basal or stress-induced mitophagy are observed in LRRK2 models^68–72^. These inconsistencies may be related to the particular methodology to measure mitophagy that was used, which *LRRK2* mutation was investigated and/or reflects cell-type context dependency.

However, treatment with MLi-2 or EB-42618 under conditions shown here to effectively inhibit LRRK2 phosphorylation biomarkers and rescue mtDNA damage phenotypes, failed to rescue the FCCP-stimulated mitophagy defect in LRRK2^G2019S/G2019S^ KI cells. Thus, suggesting that abrogation of mtDNA damage related to LRRK2 kinase inhibition in this acute context is not dependent upon mitophagy. This data are aligned with recent findings that impaired mitophagy and other mitochondrial parameters were not corrected by MLi-2 in R1441C LRRK2 cultures^71^, but inconsistent with pharmacological rescue of mitophagy defects observed in LRRK2 G2019S mice^68^. While our data do not support a role for mitophagy in mtDNA damage reversal during this acute phase, this certainly does not rule out a critical role for mitophagy with lengthier treatments with LRRK2 kinase inhibitors. Mitophagy is a complex, dynamic process which relies on numerous molecular steps and protein networks, most of which do not occur rapidly and require a longer time to effectively implement cellular machinery. While the rapid LRRK2 kinase mediated mtDNA damage reversal is a mitophagy independent process, future experiments with chronic LRRK2 kinase inhibition will encompass evaluating other facets of mitophagy-related pathways shown to be disrupted in LRRK2 PD model systems, including but not limited to impairment of PINK1/Parkin-dependent mitophagy signaling, aggregation and spatial arrest of damaged mitochondria to allow mitophagy initiation, and maturation of autophagosomes during mitophagy^69,70,73^.

Acidification of lysosomes is an important step for the proper accomplishment of any type of autophagic process, as important degradative lysosomal enzymes require a low pH to function^74^. The data shown here may suggest impaired lysosomal acidification and a defective (potentially delayed) trafficking of mitochondria to the lysosome associated with the G2019S LRRK2 mutation, consistent with previous reports of impairment of degradation of mitochondria engulfed in mature lysosomes and other lysosomal defects^71,75,76^. Overall, given the speed of the reversal and re-establishment of the mtDNA phenotype, mechanisms that are more rapid, such as mtDNA repair^77^, will be investigated to better understand the dynamic nature of LRRK2 dependent mtDNA damage.

Under conditions in which G2019S-LRRK2 and WT-LRRK2 are expressed equally, we found that mtDNA damage levels are significantly enhanced with the PD mutation. These results are consistent with our previous findings that expression of WT-LRRK2 had no effect on mtDNA damage in neurons^44^. These data suggest that accumulating mtDNA damage depends on LRRK2 kinase activity and not LRRK2 levels. However, since increased LRRK2 expression alone has been associated with toxicity^78^, untangling the effect of levels of LRRK2 from kinase activity per se, will be important next steps. Alternative approaches to therapeutically targeting LRRK2 with small molecules have emerged^13^. Antisense oligonucleotides (ASOs) in a preclinical proof of concept study showed that LRRK2 mRNA was reduced in the brain while sparing peripheral tissues^79^. Early phase human clinical studies are underway using an ASO targeting LRRK2 (ClinicalTrials identifier NCT03976349). The on-going and future ASO and LRRK2 kinase inhibitor targeted approaches will examine if reducing LRRK2 levels and/or kinase activity ameliorates the toxic gain-of-function effects of LRRK2 in PD patients and results in clinically relevant efficacy.

A precision medicine approach is common in many fields but has only been recently applied to neurodegenerative diseases and could in part explain the failures of this “one size fits all” approach to PD trials^80^. A precision medicine approach that accounts for an individual’s genotype and/or phenotype may pave the way for successful disease-modifying treatments. Although, this approach is not without its challenges and does not guarantee a positive outcome (ClinicalTrials.gov Identifier: NCT02906020). Biomarkers will be critical to guide patient stratification, response to treatment and disease progression. Advancing a precision medicine based therapeutic approach for LRRK2-associated PD will require integrated measures with a range of clinical and molecular parameters^81^.

## Methods

### LRRK2 cell lines and PD patient *LRRK2* mutation carriers and healthy control lymphoblastoid cells

HEK293 cells stably transfected with human LRRK2 or the G2019S variant of human LRRK2 (WT-LRRK2 and G2019S-LRRK2^20^, were maintained in an incubator at 37 °C with 5% CO_2_ and grown in Eagle’s Minimum Essential Medium (ATCC: The Global Biosource, 30-2003), 10% Gibco Fetal Bovine Serum, Qualified (Fisher Scientific, 10-437-028), and 0.5% Penicillin-Streptomycin (Corning, 30-002-Cl). Geneticin (Thermo Fisher Scientific, 10131035) was added to media with cells at a concentration of 400 μg/ml. Cells were plated at a density of 0.5 × 10^6^ in a 6-well dish and treated at ~40–50% confluency. LRRK2^G2019S/G2019S^ KI HEK293 cells were generated using a CRISPR/Cas9 genome editing approach which was described previously^32^. The LRRK2^G2019S/G2019S^ KI HEK293 and wild-type cells were maintained in DMEM/F12, GlutaMAX™ supplement (Gibco, 10565-018), Seradigm Premium Grade 10% FBS (VWR, 97068-085) and 0.5% Penicillin Streptomycin (Corning, 30-002-Cl). Cells were plated at a density of 0.3 × 10^6^ cells in a 6-well dish and treated at ~40–50% confluency. At the time of harvest, cells were collected and either protein collected or DNA extracted as described below.

For wash out experiments, LRRK2^G2019S/G2019S^ KI HEK293 and wild-type cells were plated at 500,000 cells per well and cultured for 24 h to reach ~40–50% confluency. Cultures were treated for 2 h with vehicle (DMSO) or 100 nM MLi-2 in complete culture medium. Following this treatment, “Post-treatment recovery 0 h” samples were collected immediately, or vehicle or MLi-2-containing treatment medium was washed out and replaced with fresh medium and incubated for 2 additional hours and then collected as “Post-treatment recovery 2 h” samples. Isolation, quantitation, and mtDNA damage was performed as described below.

In general, the endogenous levels of LRRK2 and phosphorylation forms of LRRK2 are very low in HEK293 cells. Therefore, we were not able to verify the LRRK2 pharmacodynamic biomarkers for target engagement in the LRRK2^G2019S/G2019S^ KI HEK293 cells with treatment with either the selective or non-selective LRRK2 kinase inhibitors. To ensure target engagement with the inhibitors as expected, we evaluated pSerLRRK2 935 and pSer1292 in the WT-LRRK2 and G2019S-LRRK2 overexpression HEK293 cells. However, to test the effects of each of the LRRK2 kinase inhibitors in an endogenous LRRK2 cell system, we utilized the LRRK2^G2019S/G2019S^ KI HEK293 cells. Thus, both the endogenous and overexpression LRRK2 systems complement each other in this regard and were included.

PD patient G2019S LRRK2 (*n* = 2) and healthy subject control (*n* = 2)-derived LCLs were obtained from the NINDS Coriell biorepository (ID numbers are ND00011, ND00264, ND01618, ND02559). There was not a statistical difference in the ages between the PD patient and healthy control subjects (*p* = 0.92). LCLs were cultured at 37 °C, 5% CO_2,_ in RPMI-1640 (Sigma-Aldrich, R8758), 15% heat-inactivated fetal bovine serum (VWR Seradigm, 97068-091) and 0.5% Penicillin/Streptomycin (Corning, 30-002-CI). Cells were passaged every 3–4 days, and passage number did not exceed 20. All in vitro experiments were repeated at least three times from distinct samples.

### LRRK2 kinase inhibitors

Multiple LRRK2 kinase inhibitors were utilized for in vitro experiments including MLi-2 and EB‐42168^20,24^. The tool compound EB-42168 was discovered as part of a small molecule discovery program aimed at identifying LRRK2 kinase inhibitors that are selective for the pathogenic G2019S LRRK2 variant^20^. MLi‐2 (synthesized at WuXi in Tianjing, China), was included as a reference inhibitor and was shown previously to exhibit similar binding affinity for both WT and G2019S LRRK2^24^. ESCAPE Bio shipped MLi-2 and EB-42168 compounds to the Sanders Lab at Duke in a blinded fashion, and only after results were analyzed and final were the identity of the compounds unblinded. MLi‐2 inhibits WT LRRK2 ~1000‐fold more than EB‐42168^20^. For all compound treatments, cells were treated for 2 h with varying concentrations of EB-42168 or MLi-2. Compounds were dissolved in DMSO at an initial concentration of 20 mM and serially diluted in DMSO and added to media to achieve a final concentration in the range of 10 nM–1 μM and the same final DMSO concentration for each solution, which did not exceed 0.1% v/v.

### Western blot analysis and antibodies

For LRRK2-WT or G2019S-LRRK2, ~1.5 million cells were pelleted and resuspended in 75 μl of lysis buffer [(RIPA Buffer (Sigma-Aldrich, R0278-50ML), protease inhibitor cocktail (Sigma-Aldrich, P8340), and Halt phosphatase inhibitor cocktail (Thermo Fisher, 78420)]. For LCLs, five million cells were pelleted and resuspended in 100 µl of lysis buffer. For both types of cells lysates were centrifuged at 10,000 × *g* after a 10 min incubation on ice, and the supernatant was collected. Protein was quantified using the DC protein assay (Bio-Rad, 5000112). In total, 50 μg of protein was loaded for HEK293 LRRK2-WT or G2019S-LRRK2 cells. Due to the differing endogenous LRRK2 levels, 40 µg of ND02559, 60 µg of ND00264, or 100 µg of ND00011 and ND01618 of protein sample was loaded. Protein samples were incubated at 100 °C for 5 min with NuPAGE Sample loading dye (Thermo Fisher, NP0007) and dithiothreitol as reducing agent. After 4–20% SDS-PAGE, the blots were blocked in 5% w/v nonfat dry milk in 1X PBST (0.05% Tween 20). For our investigations, the following primary antibodies were used: mouse anti-LRRK2 N241a (Antibodies INC, 75-253, 1:5000 for HEK293 cells and 1:2000 for LCLs), rabbit anti-LRRK2 pS935 (Abcam, ab133450, 1:10,000 for HEK293 cells and 1:2000 for LCLs), mouse anti-β-actin (Novus, 8H10D10, 1:10,000), and rabbit anti-LRRK2 pS1292 (Abcam, ab203181, 1:500). The blots were then probed with fluorescent-labeled secondary antibodies, IRDye donkey anti-mouse and anti-rabbit at 1:10,000 (LI-COR, 926-32212, 926-68072), and scanned using an Odyssey Imaging scanner (LI-COR). Fluorescence intensities were quantified using ImageStudio Lite software (LI-COR), and the signal from the protein of interest was normalized to the fluorescence intensity of either LRRK2 or β-actin. Values were averaged from at least three technical replicates within a cell line, or two biological replicates (two healthy control lines and two PD LCLs). All blots were derived from the same experiment and processed in parallel.

### DNA isolation, quantitation and mitochondrial DNA damage measurement

Cells were collected and the nuclei and mitochondria were isolated as previously described^49^. DNA was extracted from LRRK2-WT and G2019S-LRRK2 cells, and wild-type and LRRK2^G2019S/G2019S^ KI cells using QuickGene DNA Whole Blood Kit L (Autogen, fk-dbl). PD patient LRRK2 G2019S and healthy subject control-derived LCL DNA was extracted using the QuickGene DNA Tissue Kit L (Autogen, fk-dtl), utilizing a semi-automated system (Autogen, QuickGene-610L). For the Autogen system, the pellet was resuspended in 2 ml of 1X PBS, and the standard manufacturer’s protocol was conducted as if the cell suspension was whole blood. DNA was eluted with EDTA-free buffer, and quality was assessed using a Spectradrop microvolume microplate (Molecular Devices). Double-stranded DNA was quantified using Quant-iT Picogreen dsDNA assay (Thermo Fisher) as previously described^49^.

DNA damage in the mitochondrial genome was measured utilizing Mito DNA_DX,_ an assay that we recently developed and described^39^, currently the most robust way of measuring damage in mtDNA^39,82^. The Mito DNA_DX_ assay combines optimized polymerase chain reaction (PCR) parameters using a high-fidelity DNA polymerase and a fluorescent dye that binds to double-stranded DNA that permits the real-time quantification of DNA damage in distinct loci in a 96-well platform. The premise of the Mito DNA_DX_ assay involves amplification of a specific mitochondrial PCR fragment in which less PCR product will be amplified when mtDNA damage or lesions block the ability of the DNA polymerase to replicate. As such, mtDNA damage or mtDNA repair intermediates that slow down or impair DNA polymerase progression will be detected. Therefore, equal amounts of sample DNA amplified under identical conditions can be directly compared across conditions to determine the mtDNA lesion frequency. Mitochondrial DNA lesion frequency was performed as previously described^39^. Briefly, 15 ng of DNA was used to amplify long or short amplicons of the mitochondrial genome (as determined by primer sets). The amount of amplification is directly proportional to the number of undamaged DNA templates. Average lesion frequency is calculated as described^39^. Results are presented with the values in experimental samples relative to control samples (normalized to 1 lesion per 10 kb). PCR reactions included KAPA Long Range HotStart DNA Polymerase (KAPA Biosystems)^49^. Each biological DNA sample was analyzed in technical triplicate. Limitations of the Mito DNA_DX_ assay have been recently discussed^39^.

### Quantification of mitophagy in vitro

Thirty-five mm cell culture dishes (Cellvis, D35-20-1.5-N) were coated with poly-D-lysine (Sigma-Aldrich, P7280-5MG) for 5 min, washed with cell culture grade water (Sigma-Aldrich, W3500), and allowed to dry for at least 1 h before plating. LRRK2^G2019S/G2019S^ KI HEK293 and wild-type cells were passaged as described above and plated at 300,000 cells per well on poly-D-lysine-coated dishes. Cultures were grown for 24 h at 37 °C and then pre-treated for 2 h with 100 nM MLi-2, EB-42618, or DMSO. Following this, cells were washed three times with serum-free medium, and a working solution of Mtphagy dye (Dojindo, MD-01) was prepared in serum-free medium according to manufacturer protocols. Cultures were incubated with Mtphagy dye for 30 min, followed by three washes with serum-free medium. Complete medium was used to prepare a 20 µM FCCP (Abcam, ab120081) or DMSO control treatment solution, which was used to treat cells for 3 h. Following treatment, cells were then washed 3 times with serum-free medium, and a working solution of Lyso dye (Dojindo, MD-01) was prepared in serum-free medium and used to treat cells for 30 min. Complete medium lacking phenol red was prepared for use in confocal live cell imaging by combining phenol red-free DMEM/F-12 (Fisher Scientific, 21-041-025), FBS (VWR, 97068-085), and penicillin-streptomycin (VWR, 45000-652). Following Lyso dye treatment, cultures were washed three times with phenol red-free medium, followed by adding fresh phenol red-free medium to cell culture dishes. MitoBright LT Deep Red (Dojindo, MT12-12) was diluted in growth medium to prepare a 0.1 μmol/l solution and was used to treat cells for 15 min for total mitochondrial staining. Live cell imaging (5% CO_2_, 37 °C) was performed using a Zeiss LSM 880 Confocal Laser Scanning Microscope with Zen Black software. On average, ~70 cells were analyzed for each condition per each experimental replicate.

Following imaging, files were processed using Zen Blue software, and TIFF files of each channel were obtained for analysis. ImageJ was used to obtain readouts for image analysis. All images were converted to 8-bit format. For each respective image, the Lyso dye channel was used to create a mask delineating each lysosomal focus as a region of interest (ROI). Thresholding parameters were adjusted between biological replicates as required to ensure that the Lyso dye ROI encompassed all lysosome foci appropriately, and this was used to create a mask. This was then applied to the corresponding Mtphagy dye channel image, allowing collection of Mtphgy fluorescence intensity readouts for each lysosomal focus. Outliers were removed using Prism software (GraphPad) with the coefficient of removal set to 0.2%. Data was thresholded to exclude lysosomes not containing mitochondria by excluding fluorescence values with intensities equal to or below background levels. The number of ROIs remaining after thresholding was used to quantify lysosomes containing fluorescing mitochondria (mitolysosomes). Fluorescence intensity values for all mitolysosomes per condition per biological replicate were averaged to obtain a value of mitophagy fluorescence intensity per mitolysosome. For each replicate, all values of average mitolysosome fluorescence intensity were normalized to wild-type (vehicle treated) levels for the given replicate.

### Statistical analyses

Data were analyzed in Prism software (GraphPad). Data were analyzed by either unpaired, two-tailed Student’s *t* test or ANOVA with a Tukey or Bonferroni’s post hoc analysis. *p* values < 0.05 were considered significant. For all graphs, the bars represent mean ± standard error of the mean (SEM). The SEM was used to calculate the error bars as an inferential measure of how variable the mean value expressed on the graph will be if the assay is replicated multiple times. Blinding to treatment group and analysis was implemented when possible.

### Reporting summary

Further information on research design is available in the Nature Research Reporting Summary linked to this article.

### Supplementary information

Supplemental Material

Reporting summary

## Data Availability

The data that support the findings of this study does not present restrictions and are available upon request to the corresponding author.
