# Supplementary material for: G2019S selective LRRK2 kinase inhibitor abrogates mitochondrial DNA damage
Source: NPJ Parkinsons Dis. 2024 Mar 1;10:49. doi: 10.1038/s41531-024-00660-y (PMC10907374; doi:10.1038/s41531-024-00660-y)
Supplement: Supplementary file 1 — Supplemental Material [file 41531_2024_660_MOESM1_ESM.pdf]

G2019S selective LRRK2 kinase inhibitor abrogates mitochondrial DNA damage
Supplementary information

Authors: Nicholas Pena[1,2], Tara Richbourg[1,2], Claudia P. Gonzalez-Hunt[1,2], Rui Qi[1,2], Paul Wren[3], Carrolee Barlow[3], Natalie F. Shanks[3], Holly J. Carlisle[3], and Laurie H. Sanders[1,2*]

[1] Departments of Neurology and Pathology, Duke University School of Medicine, Durham, NC, 27710.

[2] Duke Center for Neurodegeneration and Neurotherapeutics, Duke University, Durham, North Carolina

[3] Prior employees of ESCAPE Bio, Inc., South San Francisco, California, 94080, United States

*To whom correspondence should be addressed laurie.sanders@duke.edu L.H. Sanders, PhD.

Duke University Medical Center
Room 5128, MSRBIII
3 Genome Ct
Durham, NC 27710
Office Tel: (919) 613-3890

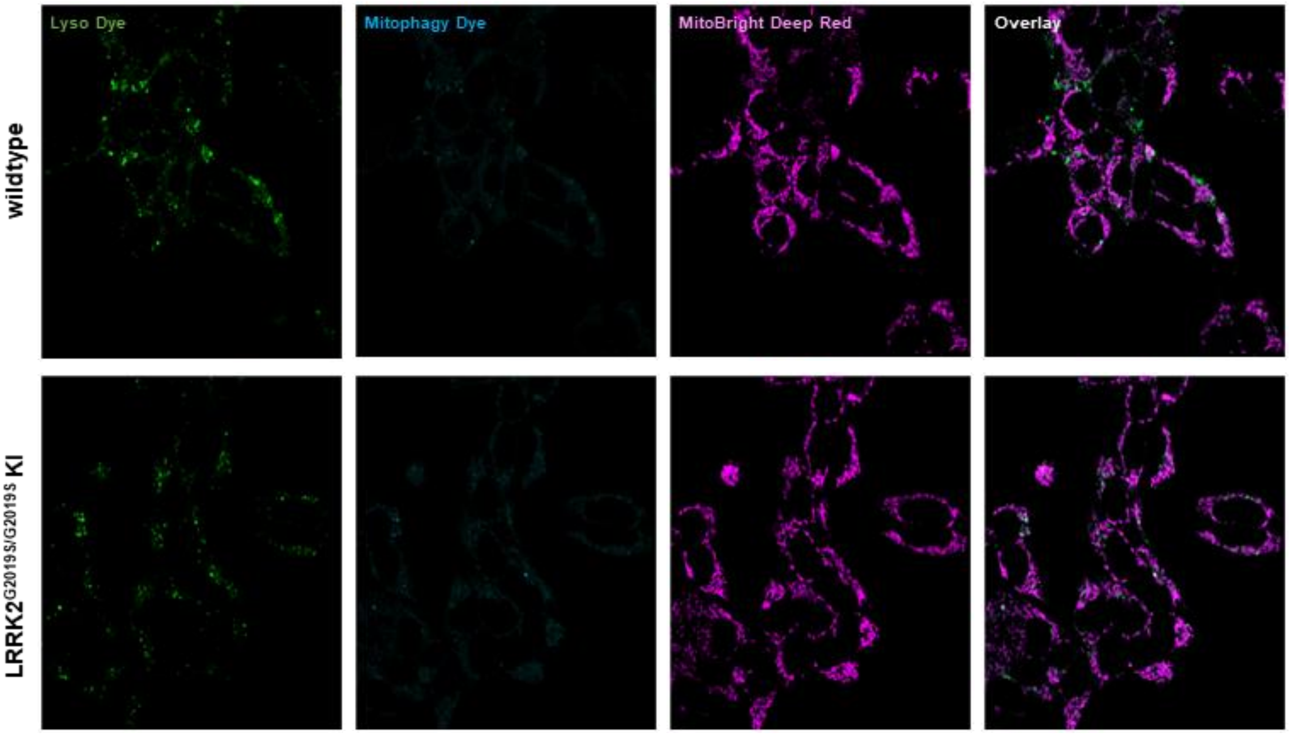

Supplemental Figure 1. Mitochondrial and lysosomal dyes used to measure mitophagy. Wild-type or LRRK2$^{G2019S/G2019S}$ KI cells were co-labeled with the lysosome and mitophagy dye. Due to the properties of the mitophagy dye which fluoresces weakly at cytosolic pH and is therefore weakly fluorescent, the mitochondrial network was visualized using the total mitochondrial stain, Mitobright LT Deep Red.
